# Neuralgia and Rheumatism Treated by Cold Draughts after Sweating

**Published:** 1850-04

**Authors:** 


*Neuralgia and Rheumatism treated by cold draughts after sweating.*—At Bellevue, near Paris, there is a fine establishment, in which everything of practical value connected with "the water-cure"—be it hot or cold—is applied to the treatment of various obstinate affections. The advantages obtained from a rational employment of several powerful agents, as distinguished from the empirical use of one alone, are very great. They were pointed out in an excellent Memoir which Mr. Fleury presented at the last meeting of the Academy of Sciences. The author selected forty-six cases, observed at the establishment during the last four years, and from their results deduced the following conclusions :

*Five* patients, laboring under attacks of acute neuralgia from four to fifteen days, (facial, intercostal, sciatic,) were cured by one to three applications of the cold douche, both general and local, employed after the use of the dry stove, which had produced copious transpiration. Here the revulsive action of heat followed by cold was much more energetic than that of flying blisters, or the cautery.

*Eleven* patients, attacked by acute muscular rheumatism, fixed in its seat and very severe, were rapidly cured in the same manner.

In *four* cases of obstinate neuralgia, which had resisted every known method of treatment for four to ten years, a cure was obtained by cold douches (general and local,) sometimes preceded by the use of the hot-air bath. The duration of the treatment varied from one to six months, and its average was three months. *Three* patients, who for five to fifteen years had presented, in the most marked degree that *ensemble* of symptoms known under the title of "nervous accidents," and who had been reduced by them to the lowest state, in spite of medical art, were cured in the same manner. Here, however, the treatment was continued from seven to eighteen months, and the average duration was more than a year.

Finally, in twenty-three cases of chronic muscular rheumatism, which had resisted every species of treatment, and the most celebrated mineral waters of Europe, the cold douches after sweating effected complete cures. The average time of treatment was four months ; the minimum one month ; the maximum seven.

Here, it must be confessed, we have a rational method of treatment, applied according to the rules of art, and as successful as the miracles of Hydropathy.—*Lond. Med. Times.*

